# Optimizing Pediatric Patient Comfort: A Study of Moisture Isolation Techniques During Dental Treatments

**DOI:** 10.3390/children13020233

**Published:** 2026-02-06

**Authors:** Maham Masud Khan, Jose Garcia, Marzia Mustamand, Xinbin Gu, Indra Mustapha, LaToya Barham

**Affiliations:** College of Dentistry, Howard University, District of Columbia, Washington, DC 20059, USA

**Keywords:** pediatrics, salivary cortisol, endocrinology, pediatric dentistry, dental isolation techniques, cotton roll isolation, DryShield, Isolite system

## Abstract

**Background:** Effective dental isolation is crucial for successful restorative procedures in pediatric patients; however, its potential impact on patient stress remains underexplored. This investigation comprised two independent pilot sub-studies evaluating salivary cortisol responses to dental isolation techniques: one comparing cotton roll isolation (CRI) and the Isolite system (IS), and a second comparing cotton roll isolation (CRI) and the DryShield isolation system (DSI). The sub-studies were reported together due to a shared clinical context and outcome measure. **Methods:** Pediatric patients underwent sealant placement using CRI, IS, or DSI, depending on sub-study assignment. Salivary cortisol samples were collected for each procedure. In the CRI–IS sub-study, pulse rate was recorded at three time points, and participants completed subjective preference surveys. Cortisol analyses were conducted separately within each sub-study, with pulse rate and preference outcomes evaluated only for the CRI–IS cohort. **Results:** DSI produced a significant increase in salivary cortisol from pre- to post-procedure compared with CRI (*p* = 0.0001), indicating a higher acute stress response. In contrast, CRI and IS did not differ significantly in cortisol levels, but heart rate did significantly increase from pre- to post-procedure when CRI was used (*p* = 0.035). Of the 15 participants in the CRI–IS comparison, 9 provided subjective feedback, with most preferring the IS. Gender was not associated with differences in stress markers in either sub-study. **Conclusions:** These findings suggest that while CRI and IS produce comparable physiological stress responses, DSI may be associated with heightened cortisol reactivity. Although IS was subjectively preferred, biological stress measures showed no definitive difference from CRI. Clinicians may therefore select CRI or IS based on clinical judgment and patient comfort, while considering the potential for increased stress when using DSI in pediatric populations.

## 1. Introduction

Isolation methods constitute a fundamental aspect of high-quality and long-lasting dental therapy and treatments [1]. The literature indicates that the use of isolation techniques—including rubber dams, cotton rolls, and the Isolite system—results in improved longevity of restorations and operator efficiency, as well as enhanced bonding of restorative materials [2,3,4]. Although the rubber dam is regarded as the “ideal tool” for isolation, there is ongoing controversy in the current literature regarding its efficacy; several comparative studies evaluating rubber dam isolation (RDI) against alternative isolation techniques have reported no statistically significant differences in clinical outcomes [5,6,7,8]. However, other studies state that the rubber dam continues to be the most effective isolation measure [9,10].

The Isolite system (IS) represents a recent innovation that functions as a combined device for suction, retraction, illumination, and isolation [11]. Although the current literature on the efficacy of Isolite systems is limited, existing publications support its effectiveness as an isolation device [12,13]. Additionally, cotton rolls remain a widely utilized and convenient method of isolation employed by many practitioners [7]. While various isolation methods are available for clinicians to choose from at their discretion, research generally emphasizes the importance of utilizing isolation techniques to achieve high-quality treatment outcomes [6]. In pediatric patients, multiple factors must be carefully considered to ensure the delivery of high-standard care that fosters effective communication and trust between the patient and the provider [14]. Maintaining low stress levels in pediatric patients is fundamental to facilitating a successful appointment experience [15].

An additional isolation system known as DryShield (DS) has recently been introduced, bearing similarities to the Isolite system by integrating functions such as fluid evacuation, tongue and cheek retraction, and bite block provision [13]. Nonetheless, DS differentiates itself through its autoclavable nature and the absence of an illumination feature. Its distinctive design enables it to effectively isolate and evacuate half of the oral cavity at a time [16]. Despite the limited literature on DryShield Isolation (DSI), one study of 28 pediatric patients (ages 9–15) compared DSI and rubber-dam isolation (RDI), and yielded noteworthy results [16]. Researchers observed that DSI was associated with reduced chair time during fissure sealants; however, children did report that it was noisier [16]. Older children also reported more tongue pressure and pain regardless of the system used [16].

The American Academy of Pediatric Dentistry (AAPD) guidelines continuously emphasize the importance of creating a positive dental experience for pediatric patients [14]. Limited research currently exists on the effects of dental isolation techniques on stress levels in pediatric patients. According to the World Health Organization (WHO), stress can be defined as a state of worry or mental tension caused by a difficult situation [17]. Stress can be categorized as either physiological or psychosocial, with physiological stress arising from factors such as trauma, pain, or illness [18]. Acute or chronic stress can be measured and quantified through biomechanical or physiologic markers such as heart rate, blood pressure, and hormone levels [19,20]. The cortisol hormone is a stress mediator that circulates in the body in response to induced stress. Serum and salivary cortisol levels reveal acute changes in response to stress [21]. Ultimately, the concomitant biomarker of stress is cortisol, as regulated by the hypothalamic–pituitary–adrenal (HPA) axis stress response activity [22]. During the stress response, salivary cortisol and α-amylase enzymes are produced respectively by the hypothalamus–pituitary–adrenal (HPA) axis and the induction of the sympathetic adrenomedullary (SAM) system [23]. Therefore, the biological impact of stress can effectively be measured by salivary cortisol levels [23]. The use of salivary cortisol is a potential diagnostic tool for detecting stress levels [23].

This study investigates whether the use of the DryShield system, the Isolite system, or cotton roll isolation during sealant placement affects intra-appointment salivary cortisol levels in pediatric patients. Additionally, the study examines both pulse rates and subjective patient experiences related to the use of cotton rolls and the Isolite system. The overall objective of this investigation is to evaluate the impact of these isolation methods on both physiological and subjective indicators of stress within pediatric patients from the Howard University Pediatric Dental Clinic.

To address this objective, the investigation was conducted as two related but methodologically distinct sub-studies: one comparing CRI and IS (CRI–IS) and a second comparing CRI and DSI (CRI–DSI). These sub-studies were performed in separate participant cohorts with differences in data collection protocols; however, they were combined into a single report due to their shared clinical context, outcome measure (salivary cortisol), and focus on pediatric dental isolation techniques. Each sub-study was analyzed independently, and no direct statistical comparisons were made between IS and DSI.

Understanding how isolation techniques influence pediatric stress responses is essential for informing treatment planning, behavior management, and the delivery of high-quality care [14]. However, it is beyond the scope of this research to recommend specific isolation methods for dental procedures. Rather, this study aims to provide a comprehensive evaluation of stress levels and patient preferences associated with different isolation techniques. The findings may be used at the reader’s discretion to guide clinical decision-making to promote a positive experience for pediatric patients.

## 2. Materials and Methods

### 2.1. Research Design

This investigation consisted of two related sub-studies, conducted independently but reported together due to their shared objective of evaluating salivary cortisol responses to dental sealant placement in pediatric patients. Participants were enrolled in only one sub-study and were not exposed to all three isolation techniques. All participants had prior dental experience. Each sub-study was analyzed separately within its own cohort, with a single operator (2-handed) performing all dental procedures.

Sub-study 1 (CRI–IS; n = 15) compared cotton roll isolation (CRI) and the Isolite system (IS), while Sub-study 2 (CRI–DSI; n = 22) compared CRI and the DryShield isolation system (DSI). The sample size for Sub-study 2 was determined using a priori power calculations. Convenient sampling was implemented for both sub-studies.

Both sub-studies employed within-participant crossover designs in which the order of isolation techniques was randomized. In the CRI–IS sub-study, a split-mouth crossover design was used, with each participant receiving one isolation technique per visit. The alternate isolation technique was applied at a subsequent visit. In the CRI–DSI sub-study, a within-participant crossover design was implemented across two visits, with each participant receiving one isolation technique per visit applied to a single quadrant. The sequence in which isolation techniques were applied across visits was randomized.

For each participant, the order of isolation techniques (i.e., which technique was applied first) was determined immediately prior to the procedure using a coin toss, with “heads” indicating CRI and “tails” indicating the alternative isolation technique. The study coordinator, who was independent of outcome assessment, performed the coin toss and revealed the assigned sequence at the time of treatment, ensuring allocation concealment. Randomization applied to the sequence of techniques only and did not involve random assignment of techniques to specific quadrants or sides of the mouth.

**Figure 1:**
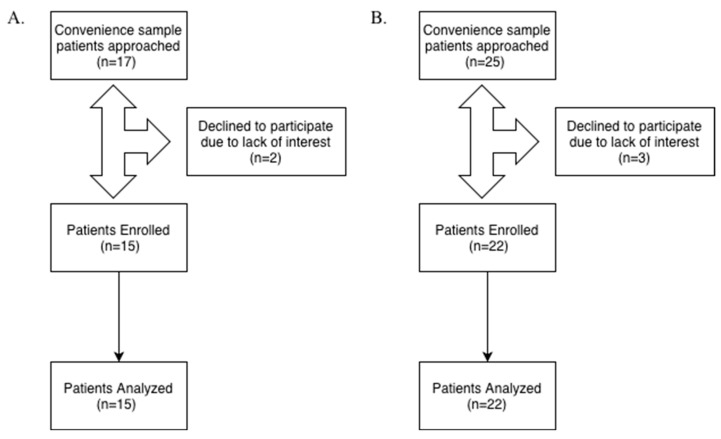
Participant flow diagram for sub-studies: (**A**) Flow of participants through recruitment, enrollment, and analysis for CRI-IS sub-study; (**B**) Flow of participants through recruitment, enrollment, and analysis for CRI-DSI sub-study.

Although operator blinding was not feasible due to the nature of the interventions, all analyses of salivary cortisol and pulse rate were performed by personnel blinded to the assigned isolation technique. Figure 1 depicts the flow of participants through recruitment, enrollment, and analysis for each sub-study.

When comparing CRI and IS, cortisol levels were measured with kits from an external supplier—SalivaBio Collection Aid—and tested at Howard University using the Salimetrics^®^ High Sensitivity Salivary Cortisol Enzyme Immunoassay Kit (Item No. 1-3002) (State College, PA, USA), following the manufacturer’s instructions. In parallel, radial pulse rates were recorded manually immediately after applying the intraoral isolation. For the comparison between CRI and DSI, cortisol levels were assessed using Labcorp’s Salivary Hormone Collection Kit (Burlington, NC, USA), sent directly to their lab for hormone analysis. This approach ensures that each comparison utilizes the analytical platform most compatible with the corresponding collection kit, thereby maintaining assay validity, optimizing sample integrity, and ensuring consistency in the laboratory methods used for each comparison.

Trial registration: These sub-studies were conducted as exploratory pilot RCTs and were not registered in a public clinical trial registry.

### 2.2. Sample Size and Gender Distribution

For both sub-studies, participants were selected from the Pediatric Dental Department at Howard University College of Dentistry and required to be between 6 and 18 years of age. The CRI–IS sub-study was designed as a pilot exploratory analysis, enrolling a convenience sample of 15 pediatric patients, including 11 females and 4 males.

In the CRI–DSI sub-study, a priori power and sample size calculations were performed using the OpenEpi (Version 3.01) sample size calculator, resulting in a target sample of 22 pediatric patients with an intended 1:1 male-to-female ratio to allow for evaluation of potential gender-related differences in outcomes. Although patients up to 18 years of age were eligible for enrollment, all participants who completed the study were between 6 and 12 years old, as older eligible patients did not participate during the study period.

### 2.3. Inclusion and Exclusion Criteria

For both sub-studies, eligible participants were required to be between 6 and 18 years of age. For the CRI-IS sub-study, participants required bilateral sealants, enabling the use of both isolation techniques. For the CRI-DSI sub-study, participants were required to have at least one dental sealant indicated for the first or second permanent molars in two quadrants, allowing for the application of both isolation techniques. Individuals with a history of negative dental experiences or those with special health care needs were excluded from all components of the study.

### 2.4. Data Collection: Cotton Roll Isolation and the Isolite System

**Figure 2:**
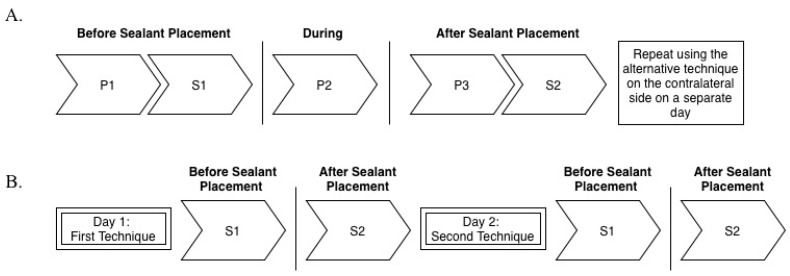
Data Collection Sequence: (**A**) Collection Sequence: Cotton Roll Isolation and the Isolite System; (**B**) Collection Sequence: Cotton Roll Isolation and DryShield Isolation.

For the comparative assessment of CRI and IS, saliva was initially collected using the SalivaBio Collection Aid; however, inadequate saturation of the cotton swabs prompted a switch to the passive drool method. Patients were seated in the operatory and engaged in brief conversation to minimize anxiety. A pulse oximeter was placed on the index finger, and a baseline pulse rate was recorded (P1). Unstimulated whole saliva was then collected via passive drool into sterile polypropylene vials using the SalivaBio Collection Aid (S1). A second pulse rate was recorded after rinsing off the etchant (P2). Upon completion of sealant placement, patients were returned to an upright position, pulse rate was recorded again (P3), and a second saliva sample was collected (S2). Refer to Figure 2A for a visual summary of the data collection sequence. Patients were then dismissed and scheduled for the contralateral side, which was treated using the alternate isolation method following the same protocol. All collected samples were immediately stored in a freezer according to the manufacturer’s instructions. After completing appointments with both isolation methods (Isolite and cotton rolls), patients were asked to indicate their preferred method and provide the reason for their preference.

Salivary cortisol concentrations were quantified using the Salimetrics^®^ High Sensitivity Salivary Cortisol Enzyme Immunoassay Kit (Item No. 1-3002), following the manufacturer’s instructions. This competitive ELISA employs monoclonal antibodies against cortisol and a cortisol–horseradish peroxidase conjugate. High and low cortisol controls provided with the kit were included in each run. All samples and controls were assayed in triplicate, and the average optical density was used for calculations. Absorbance was measured at 450 nm with a reference filter of 490–492 nm. Results were calculated using a four-parameter nonlinear regression curve, and cortisol concentrations were interpolated from the standard curve. Samples exceeding 3.0 µg/dL were diluted with assay diluent and reanalyzed, with final concentrations adjusted by the appropriate dilution factor.

### 2.5. Data Collection: Cotton Roll Isolation and DryShield Isolation

When conducting the comparative analysis of CRI and DSI, patients received at least one dental sealant procedure on the first or second permanent molars in two quadrants. Saliva samples were collected at four stages of the procedure: pre-treatment on day one (S1), after placement of the first isolation method (S2), pre-treatment on day two (S1), and after placement of the second isolation method (S2). A one-day interval between visits was incorporated to allow salivary cortisol levels to return to baseline. A schematic of this data collection process is provided in Figure 2B.

The salivary collection kits used for cortisol analysis were procured from LabCorp and submitted to their laboratory for testing. Each kit contained a centrifuge tube and an absorbent cotton pad. In accordance with LabCorp guidelines, participants refrained from eating or drinking for at least 30 min prior to collection, and the oral cavity was inspected to ensure the absence of blood contamination. After placement of the assigned isolation technique, the LabCorp cotton pad was positioned on the floor of the mouth for a minimum of 90 s, then removed and returned to the centrifuge tube. Samples collected on the first day were refrigerated until the second-day samples were obtained, after which both were delivered to a LabCorp facility for analysis.

### 2.6. Statistical Analysis

Within each sub-study, comparisons of salivary cortisol and heart rate between isolation techniques were performed using paired *t*-tests. Normality of the paired differences was assessed using both the D’Agostino & Pearson omnibus test and the Shapiro–Wilk test, and all comparisons met the assumption of approximate normality. Repeated measures within participants were accounted for by performing analyses within each sub-study, with each participant acting as their own control. Effect sizes (Cohen’s d) and 95% confidence intervals were calculated for all paired comparisons to quantify the magnitude and precision of differences. The sub-studies were analyzed separately, and direct statistical comparisons between IS and DSI were not performed.

## 3. Results

### 3.1. Comparative Analysis: CRI and DSI

**Figure 3:**
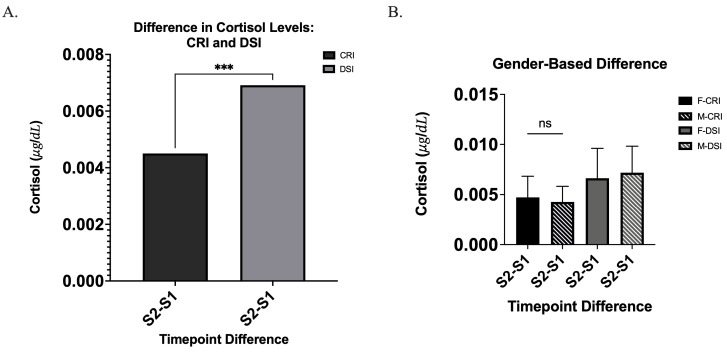
(**A**) Cortisol level differences between CRI and DSI. Bar graph representing the mean change in salivary cortisol concentrations (S2–S1) for the Cotton Roll Isolation (CRI) and the DryShield Isolation (DSI). The DSI produced a significantly greater increase in cortisol levels (µg/dL) compared with CRI (*** *p* < 0.001). (**B**) Gender-based comparison of cortisol differences for each technique. Cortisol changes (S2–S1) are shown for females and males undergoing CRI and DSI. No significant differences were observed between genders within either technique (ns).

A significant difference in cortisol response was identified between the CRI and DSI techniques (Figure 3A). Participants undergoing the DSI method (mean = 0.0069 μg/dL) exhibited a greater increase in cortisol levels from pre- to post-procedure compared to those treated with the CRI technique (mean = 0.0045 μg/dL), and this difference was statistically significant (*p* = 0.0001, *d* = 1.002) (Figure 3A). The mean increase in cortisol was 0.00241 μg/dL (95% CI: 0.00134 to 0.00347) (Figure 3A). Conversely, no significant differences in cortisol levels were detected between genders (*p* = 0.65, *d* = 0.14 (Figure 3B). The mean difference in cortisol was −0.0004545 μg/dL (95% CI: −0.002629 to 0.001720) (Figure 3A). These findings suggest that the DSI technique may elicit a greater acute stress response relative to the CRI technique in pediatric patients. However, findings of this sub-study should be interpreted cautiously, as its pilot design and use of convenience sampling limits generalizability.

### 3.2. Comparative Analysis: CRI and IS

**Figure 4:**
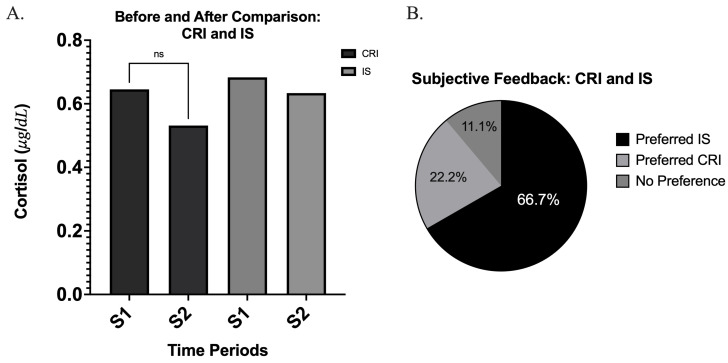
(**A**) Before-and-after salivary cortisol comparison for CRI and IS. Mean cortisol concentrations (µg/dL) are shown for each technique at two time points (S1 = pre-procedure, S2 = post-procedure). No significant difference was observed between S1 and S2 for either technique (ns). (**B**) Subjective preference for CRI and IS. Pie chart illustrating participants’ reported preferences following both procedures. The majority preferred the Isolite System (66.7%), while 22.2% preferred the Cotton Roll Isolation (CRI), and 11.1% reported no preference.

No meaningful conclusions could be drawn regarding differences between the CRI and IS techniques. Although IS was subjectively preferred and showed a slight decrease in cortisol levels, this reduction was not statistically significant (*p* = 0.815, *d* = 0.072) (Figure 3A,B). The mean difference in cortisol was −0.04972 μg/dL (95% CI: −0.5115 to 0.4120) (Figure 4A). Similarly, the CRI technique demonstrated a modest decrease in cortisol levels, but this change was also not statistically significant (*p* = 0.28, *d* = 0.30) (Figure 3A). The mean difference in cortisol was −0.1137 μg/dL (95% CI: −0.3320 to 0.1046) (Figure 4A).

**Figure 5:**
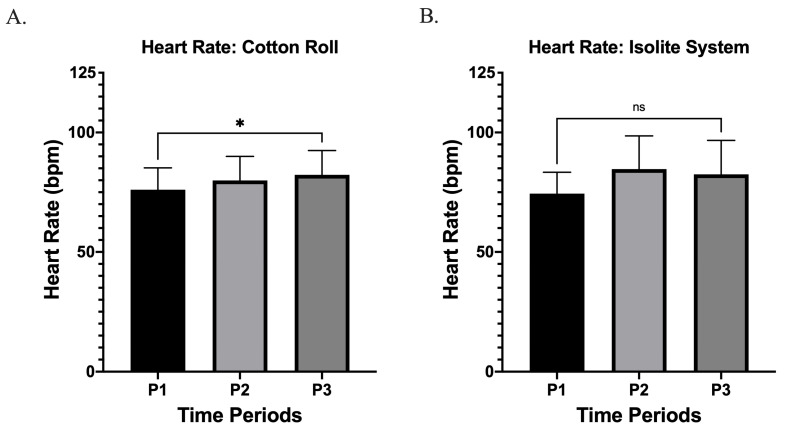
(**A**) Heart rate during Cotton Roll Isolation (CRI). Mean heart rate (bpm) recorded at three time points (P1, P2, P3) showed a statistically significant increase across the procedure (* *p* < 0.05). Error bars represent standard deviation. (**B**) Heart rate during the Isolite System (IS). Mean heart rate at P1, P2, and P3 showed no significant changes throughout the procedure (ns). Error bars represent standard deviations.

Of the 15 participants, 9 provided subjective feedback, and among these patients, IS was preferred most (Figure 4B). Heart rate increased significantly from pre- to post-procedure when cotton roll isolation (CRI) was used (*p* = 0.035, *d* = 0.60) (Figure 5A). The mean increase in heart rate was 6.20 bpm (95% CI: 0.5131 to 11.89) (Figure 5A). However, no statistically significant change (*p* = 0.116, *d* = 0.55) was observed with IS, the mean increase in heart rate was 8.10 bpm (95% CI: −2.459 to 18.66) (Figure 5B).

Overall, this sub-study indicates that while patients subjectively favored the IS technique, salivary cortisol levels demonstrated that neither method produced a significant acute stress response. Although heart rate evaluation demonstrated a statistically significant increase from pre- to post-procedure, this change may not necessarily reflect an acute stress response, as heart rate is influenced by multiple physiological and contextual factors and may not directly reflect acute stress. Furthermore, the generalizability of findings from this sub-study is limited, as it was conducted as a pilot investigation using convenience sampling. Accordingly, clinicians may opt for either CRI or IS based on their own clinical judgment and patient preference.

## 4. Discussion

This study evaluated the influence of three dental isolation techniques—cotton roll isolation (CRI), DryShield isolation (DSI), and the Isolite system (IS)—on salivary cortisol levels among pediatric patients undergoing sealant placement, with additional analysis of pulse rate and subjective patient preferences for the CRI and IS techniques. Given the important role stress management and patient comfort play in pediatric dental care, examining the impact of these isolation methods on physiological and perceived stress offers important clinical implications [14].

The findings from the comparison between CRI and DSI demonstrated a statistically significant increase in salivary cortisol associated with the DSI technique. This indicates that DSI may induce a greater acute stress response than CRI in pediatric patients. One possible explanation relates to device characteristics: DSI includes features such as continuous suction, tongue retraction, and a bite block, which are designed to increase efficiency but may feel intrusive or unfamiliar to younger patients [13,16]. Although the literature on DSI is limited, existing studies similarly note that pediatric patients report discomfort related to the noise that the device emits [16]. The current results align with these observations and reinforce the importance of incorporating physiological stress markers when assessing new isolation devices for pediatric patients.

In contrast, the sub-study between CRI and IS revealed no statistically significant differences in cortisol levels. Both techniques produced slight reductions in cortisol, but neither change reached statistical significance. While heart rate increased significantly when utilizing CRI, this parameter alone does not provide a definitive indicator of acute psychological stress [24]. Multiple physiological and contextual factors influence heart rate, and its relationship with subjective stress is inconsistent, underscoring the importance of evaluating stress using additional biomarkers such as cortisol or heart rate variability [24,25]. Subjective feedback favored the IS, with 9 out of 15 participants providing feedback, and the majority indicating a preference for IS. This preference may stem from ergonomic features of the Isolite system, such as improved suction, increased comfort due to reduced need for manual retraction, or perceived procedural efficiency [11,12]. While heart rate changes appeared to align with subjective patient feedback, the absence of corroborating changes in salivary cortisol limits the ability to infer a definitive clinical outcome.

Interpretation of these findings should be approached cautiously, as both sub-studies were designed as a pilot investigation and relied on convenience sampling, which limits the generalizability of the observed effects. The lack of corroborating changes in physiological stress markers in this sub-study indicates that subjective comfort did not necessarily translate into definitive reductions in acute stress. This supports prior research demonstrating that subjective dental experiences do not always correlate with biological stress responses in pediatric populations [26].

The absence of significant gender differences adds to the existing literature, suggesting that for routine dental procedures, gender may not substantially influence acute physiological stress responses in pediatric patients. The consistency of this finding supports the internal validity of each sub-study, indicating that the observed differences are primarily attributable to the isolation techniques themselves, rather than demographic variation.

Collectively, these results emphasize that while isolation systems integrating suction and retraction features may offer procedural advantages or subjective comfort, their impact on physiological stress is not uniform across devices. DSI may warrant additional consideration when used in younger or anxious patients due to its association with elevated cortisol response. Conversely, both IS and CRI appear to be comparably well-tolerated from a physiological standpoint, allowing clinicians flexibility in choosing between them, without concern for increased stress burden. However, recommending specific isolation techniques for clinical use falls outside the scope of this investigation, given the limited generalizability of the findings. Instead, the purpose of this study is to provide a detailed assessment of pediatric patients’ stress responses and preferences across different isolation methods, addressing a topic that remains largely unexplored due to the subtle and often implicit nature of these responses. The resulting data may inform clinical judgment at the practitioner’s discretion, ultimately enabling data-driven decisions that enhance the overall experience for pediatric patients.

This investigation also highlights the value of combining objective markers—such as salivary cortisol and pulse rate—with subjective patient feedback. Pediatric patients may not possess the accurate vocabulary or self-awareness to describe discomfort, making physiologic measures a valuable tool in evaluating treatment-related stress. Given the importance of stress management and positive dental experiences, emphasized by the AAPD guidelines, integrating both biological and subjective assessments may offer clinicians a nuanced perspective to tailor treatment more effectively [14].

### Limitations and Future Directions

While this study offers valuable insights into pediatric stress responses during dental isolation, it also has several key limitations. First, the small sample sizes—15 participants in the CRI–IS sub-study and 22 in the CRI–DSI sub-study. This sample size may limit statistical power and reduce the generalizability of the findings. Furthermore, both sub-studies served as pilot investigations employing convenience sampling; the exploratory and non-randomized sampling limits generalizability and introduces potential selection bias. Additionally, the one-day gap between appointments, although intended to allow cortisol levels to return to baseline, may not have accounted for natural fluctuations caused by daily stressors, sleep quality, or circadian rhythm. Moreover, although salivary cortisol is a well-established indicator of HPA axis activation, reliance on this single biomarker may not fully characterize the complex physiological stress response.

Despite these limitations, the study provides new insights into how modern isolation systems affect pediatric stress responses and contributes meaningful evidence to the growing body of literature in pediatric dentistry. The findings suggest that CRI and IS can be used interchangeably depending on clinician preference and patient comfort, while DSI—although effective—may need careful consideration for more stress-sensitive patients. Future research with larger sample sizes, wider age ranges, randomized sampling, and additional behavioral assessment tools would help clarify how different isolation techniques influence pediatric treatment experiences. Although all procedures in the present study were standardized to a two-handed technique, future investigations may benefit from evaluating whether differences in operator working method, such as two-handed versus four-handed dentistry, influence salivary cortisol responses in pediatric patients. Longitudinal studies examining how early experiences with various isolation devices affect future dental anxiety would also enhance the clinical relevance of this topic.

## 5. Conclusions

This study demonstrates that the choice of dental isolation technique can influence physiological stress responses in pediatric patients. Within the CRI–DSI sub-study, use of the DryShield isolation system was associated with higher salivary cortisol responses compared with cotton roll isolation, suggesting increased acute stress. In contrast, findings from the pilot CRI–IS sub-study indicate that cotton roll isolation and the Isolite system elicit comparable physiological stress effects, despite a subjective preference for the Isolite system among participants.

Although these findings derive from two related but methodologically distinct sub-studies, their combined interpretation underscores the importance of selecting isolation methods that balance clinical efficiency, patient comfort, and stress management. Consideration of both objective physiological markers and subjective patient experiences may support more positive pediatric dental encounters and inform clinical decision-making in pediatric dentistry.

## Data Availability

The original contributions presented in the study are included in the article, further inquiries can be directed to the corresponding authors.

## Abbreviations

The following abbreviations are used in this manuscript:

CRI	Cotton Roll Isolation
DSI	DryShield Isolation
IS	Isolite System
RDI	Rubber Dam Isolation
DS	DryShield
AAPD	American Academy of Pediatric Dentistry
WHO	World Health Organization
HPA	Hypothalamus–Pituitary–Adrenal
SAM	Sympathetic Adrenomedullary
